# Eczema and Dermatitis
*A Paper read at the Meeting of the Bristol Medico-Chirurgical Society, on Wednesday, 10th April, 1935.


**Published:** 1935

**Authors:** Norman Burgess

**Affiliations:** Physician in Charge, Skin Department, Bristol General Hospital; Honorary Dermatologist, Albert Dock Hospital, London


### ECZEMA AND DERMATITIS.*

BY

Norman Burgess, M.D., M.R.C.P.,

*Physician in Charge, Skin Department, Bristol General Hospital ; Honorary Dermatologist, Albert Dock Hospital, London.*

---

I have chosen the subject of eczema and dermatitis for this paper in the first place because the condition to which these names are applied is so commonly met with in practice, and secondly because in the minds of many there is no clear conception of what is meant by these terms. I regret to say that even if you refer to text-books of dermatology you will fail to find complete unanimity of opinion.

In the first place, it must be realized that there is no clinical or histological difference between eczema and dermatitis. Both words are used to describe an inflammatory disease of the skin characterized by redness, papules, vesicles, pustules, scales and crusts, alone or in combination, and accompanied by itching and burning.

Histologically one finds dilatation of the blood-vessels and perivascular round-celled infiltration in the corium, while in the epidermis there is œdema both in and between the cells. The cells are pushed apart, the inter-cellular fibrils rupture and microscopic vesicles

* A Paper read at the Meeting of the Bristol Medico-Chirurgical Society, on Wednesday, 10th April, 1935.

are formed. These may increase in size and become pushed up to the surface, where they are visible to the naked eye.

If the reaction is sufficiently intense the vesicles rupture and weeping occurs. The serum may dry into crusts, and when the horny layer is reconstituted abnormal development of the cells may occur, leading to the formation of scales. Although these changes are present to some extent in all cases, one or more may predominate ; there are, therefore, several clinical forms of the disease.

*Erythematous type.*—This condition is most commonly found on the face and flexures, and is characterized by dry, red, itching patches which tend to spread and coalesce. The condition may become scaly and infiltrated, or weeping may occur.

*Papular type.*—This form is usually seen on the trunk and limbs, and itching is a prominent feature. In the acute cases the papules develop later into vesicles, while in the chronic cases infiltration is marked.

*Vesicular type.*—This is the most common form of the disease ; it usually begins as red patches, which become studded with vesicles. In most cases these rupture and weeping takes place. Crusts usually form, and later, when the exudation ceases, desquamation occurs. In long-standing cases the affected skin becomes thickened and dark red in colour as a result of infiltration, multiplication of the epidermal cells, and chronic vascular congestion. A condition known as eczema rubrum may develop on the legs, the skin is red and glazed, and there is a certain amount of weeping. On the palms and soles the vesicles tend to reach a large size, as they cannot easily rupture owing to the thickness of the horny layer. Chronic scaly eczema or dermatitis may occur on the palms and soles, in which fissures may be present which are usually sore and tender.

*Infantile eczema.*—There are two main types of infantile eczema. In the first the condition begins on the scalp, spreads to the forehead and backs of the ears, and later to the face, trunk and limbs; while in the second form the cheeks, forehead and chin are first affected, and the scalp, trunk, and limbs may be involved later.

In the diagnosis of eczema and dermatitis, as in all skin conditions, one must look for the primary lesions, which will be found in the most recently-affected parts. One must then see what evolutionary changes are taking place, *e.g.* rupture of vesicles, weeping, crust formation, etc., and finally note any secondary changes, such as impetiginization, excoriation or lichenification. Since we have seen that clinically and histologically there is no difference between eczema and dermatitis, you may well ask why two terms are used to describe the same condition, and what, if any, difference exists between them.

The difference in nomenclature is based on ætiology, which must now be considered.

All cases of eczema or dermatitis are produced by the action of an irritant on the skin, this irritant being either external to the body and coming in contact with the skin surface, or present in the body and conveyed to the skin by the blood-stream.

The irritant may be regarded as the exciting factor. In all cases except traumatic dermatitis, which is produced by substances so irritating that they will produce the condition on any normal skin, *e.g.* croton oil or mustard, a further predisposing factor must be present, *i.e.* the epidermal cells must be sensitive to the irritant concerned. For example, most gardeners can handle primula plants with impunity, and only those people who are sensitive to them develop dermatitis.

We are now in a position to see how the terms eczema and dermatitis are used.

Some use the word dermatitis for all cases in which an external cause can be found, and call those in which the cause is internal or unknown eczema. Others wish to abandon the word eczema altogether, and call all cases dermatitis, while others regard as dermatitis only the traumatic cases, and use the word eczema for those cases in which sensitization of the epidermal cells is present. I personally incline to the latter view.

Unfortunately, however, the word "dermatitis" has come to have a legal significance, and when used on a panel certificate at once suggests "compensation" to the recipient, as it has come to be regarded as synonymous with occupational eczema or dermatitis.

I would therefore suggest that while accepting the latter of the three definitions I gave just now you should extend the word dermatitis to cover, in addition to the cases of traumatic dermatitis, those cases of eczema which you believe after careful consideration to be occupational in origin.

The exciting causes of eczema and dermatitis are many. Physical agents such as light, X-rays and radium may produce the condition. Many plants can cause eczema in susceptible persons, the commonest being primula obconica, chrysanthemum, and rhus toxicodendron. Certain woods, especially teak, may also cause the condition. Mineral substances are responsible for a very large number of cases, which include the important group of occupational dermatitis.

Occupational dermatitis is usually found on the hands and forearms and sometimes, in the case of dusts, on the face, genitals and ankles as well. The disease clears up on stopping work and relapses on resuming. The fact that the patient has handled the same substance for years without developing the disease before does not necessarily indicate that the eruption is not trade dermatitis, since he may have become sensitive to the substance after its long-continued use.

Many cases of eczema are caused by the action of micro-organisms on a sensitive skin, the most common being the Pityrosporon of Malassez, which produces dandruff on the scalp and seborrhœic eczema on the body, face and limbs. These cases are very commonly met with. An infection with the dandruff organisms derived from the parent or nurse is responsible for the first group of cases of infantile eczema which I described—those in which the condition begins on the scalp and later spreads to the face and body.

Of the internal irritants the most important are antigens derived from the alimentary tract, from foci of infection, and from the breakdown of epidermal cells. A common example of the latter is often seen in cases of varicose eczema. A patient who has had a chronic patch of varicose eczema on the leg for years may suddenly develop a generalized eczematous eruption on the body and limbs.

We now come to the predisposing factors in the production of eczema. In the first place the presence of nervous strain, metabolic disturbances, general ill-health and the presence of certain cutaneous dystrophies may all act as predisposing factors in the production of the diseases. These cutaneous dystrophies are :—

1. *Xerodermia.*—This consists of a generally dry skin, slight scaliness of the forehead, scalp and pretibial regions, follicular plugging on the extensor surfaces of the limbs and dryness of the palms.

2. *The seborrhœic state.*—Excessive activity of the sebaceous glands, which is most marked on the face.

3. *Hyperidrosis.*—Most marked in the axillæ, scrotum, palms and soles.

4. *Vascular stasis,* which may be either vasomotor in origin as in rosacea, or mechanical as in varicose eczema.

In many cases there seems to be an hereditary predisposition to the development of eczema and other allergic diseases, those most commonly associated being eczema and asthma. These patients usually develop in the first few months of life infantile eczema of the second type, beginning on the cheeks, which tends to continue off and on until about two years of age. At about five or six years eczema and prurigo of the flexures and round the mouth usually develop, and later the patient may suffer from asthma, urticaria, hay-fever or migraine. There is usually a family history of eczema and asthma.

In other cases a patient may become sensitive to an irritant from long-continued exposures to it. Wile, of Ann Arbor, Michigan, succeeded in making himself sensitive to rhus by daily application of the plant to his skin. In these cases, although the application of the sensitizing substance may be limited to a small area, when the patient becomes sensitive the whole of the skin is sensitized. This may be demonstrated by means of a patch test. In a sensitive person the application of a small amount of the substance to which he is sensitive for twenty-four hours will produce an area of erythema and vesicles corresponding to the shape of the patch applied. The result is the same no matter what part of the body surface is selected for the test.

We thus see that predisposition may be inherited or may be acquired. It may also be temporary or permanent.

It follows from what has been said that the investigation of a case of eczema or dermatitis will resolve itself into two main parts, an attempt to discover the irritant and the investigation of possible predisposing factors.

In the discovery of an external irritant a careful history will often give valuable clues to likely substances. The opinion so formed may be confirmed by making patch tests with the suspected substance. One should also see if the patient has an excessive amount of dandruff, or some chronic patch of infective eczema which may be giving rise to breakdown products of epidermal cells. The patient should be examined for evidence of focal infection, and when no other cause can be found a diet chart should be made in order to see if any food substance is responsible for the condition.

Possible predisposing factors must also be considered. The family history may indicate that an hereditary predisposition exists, while the presence or absence of metabolic disorders, nervous strain and cutaneous dystrophies must be noted.

### Treatment.

If the irritant is discovered its removal will usually cause the condition promptly to subside, but care must be taken that the patient is not again exposed to its action, for if he is the condition will probably recur equally promptly. Foci of infection should be removed and metabolic diseases treated. The administration of calcium salts seems to be of value in some cases, while bromides are especially useful in weeping cases. In the latter type of case I have frequently noted rapid improvement after intravenous injections of calcium or sodium thiosulphate. In cases due to absorption of toxins from the alimentary tract intestinal antiseptics should be given. In seborrhœic eczema the administration of alkalies in large doses is useful in many cases. In some cases, especially those due to absorption of breakdown productions of epidermal cells, autogenous whole blood injections are of value. Good results are also obtained by giving intradermal injections of proteose obtained from the patient's urine.

The local treatment depends on the type of eruption present rather than on the cause. In generalized cases the patient should be kept in bed. In the acute, weeping stage wet dressings should be used, lead lotion or a 1 per cent. solution of aluminum acetate if no secondary infection is present, and a 1 : 4000 solution of potassium permanganate if it is. The method of application is important. Butter muslin or lint should be soaked in the solution, wrung until it is soppy but not dripping, and wrapped round the affected part. No oiled silk should be applied. The patient should have a bowl of the lotion by the side of his bed, and should moisten the dressing frequently, so that it is prevented from sticking.

If the lesions are small and scattered calamine lotion may be used, and various drugs may be added to it if necessary. When weeping subsides pastes may be used, Lassar's paste being most useful. Sulphur or ichthyol and later tar may be added to this, the tar being gradually increased if well tolerated. In the scaly stage ointments containing salicylic acid may be prescribed.

The skin should be cleansed with oil in all stages and soap and water should be avoided. In seborrhœic eczema it is most important to treat the scalp. Lotions containing perchloride of mercury and salicylic acid or ointments containing sulphur and salicylic acid should be used. It is also important to treat the person who is the source of the infection in cases of infantile eczema of the infective type.

A most useful preparation in the treatment of infantile eczema is the crude coal tar paste suggested by White of Boston ; this should be applied in a thin smear night and morning after cleansing with olive oil. It should not be bandaged, but may be covered with a layer of talcum powder.

Localized patches of subacute and chronic eczema will usually clear up well and rapidly if a few fractional doses of X-rays are given. Cases accompanied by infiltration or lichenification which are most resistant to other methods of treatment are particularly suitable for X-ray therapy. I usually find that three or four doses of $\frac{1}{4}$ B. given at intervals of a week or ten days will clear up these cases. It is, of course, important at the same time to investigate and treat the underlying causes of the condition, otherwise a recurrence is likely to take place.

It will be seen that the causes, clinical appearance, and treatment of cases of eczema and dermatitis present many variations. Successful treatment depends on the discovery of the cause, combined with appropriate local and internal treatment.

